# Cryoablation for Small Renal Masses

**DOI:** 10.1155/2008/479495

**Published:** 2008-07-15

**Authors:** J. L. Dominguez-Escrig, K. Sahadevan, P. Johnson

**Affiliations:** ^1^Department of Urology, Freeman Hospital, Freeman Road, High Heaton, Newcastle Upon Tyne NE7 7DN, UK; ^2^Department of Urology, Sunderland Royal Hospital, Kayll Road, Sunderland SR4 7TP, UK

## Abstract

Advances in imaging techniques (CT and MRI) and widespread use of imaging especially ultrasound scanning have resulted in a dramatic increase in the detection of small renal masses. While open partial nephrectomy is still the reference standard for the management of these small renal masses, its associated morbidity has encouraged clinicians to exploit the advancements in minimally invasive ablative techniques. The last decade has seen the rapid development of laparoscopic partial nephrectomy and novel ablative techniques such as, radiofrequency ablation (RFA), high-intensity focused ultrasound (HIFU), and cryoablation (CA). In particular, CA for small renal masses has gained popularity as it combines nephron-sparing surgery with a minimally invasive approach. Studies with up to 5-year followup have shown an overall and cancer-specific 5-year survival of 82% and 100%, respectively. This manuscript will focus on the principles and clinical applications of cryoablation of small renal masses, with detailed review of relevant literature.

## 1. INTRODUCTION

Worldwide, around 208 500 new cases of renal cancer are diagnosed each year, accounting for just under a 2% of all cancers with higher incidence in more developed countries [1–3]. Regardless of its true impact on annual incidence, the widespread use of more sensitive imaging techniques (USS, CT, and MRI) has led to an increase in the number of incidentally detected renal tumors [4–7], with an estimated increased detection of asymptomatic, small renal masses by 60% in recent years [8].

In Europe, the most recent estimates of incidence of renal cancer suggest that there are 63 300 new cases annually in the EU25, accounting for nearly 3% of all cancers [9], with an estimated annual increase in incidence of approximately 2% [2, 10]. In Spain, the estimated incidence and mortality for the year 2002 were 4085 (2778 men, 1307 women) and 1644 (1093 men, 551 women) cases, respectively (FCAECC, La situacion del cancer en España. Ministerio de Sanidad, 2005).

In contrast to a historical incidence of 5% of renal tumours of less than 3 cm in size, current incidence of such tumours ranges between 10% and 40% [11, 12]. Although the natural history and biological behaviour of this “small renal mass” are yet to be understood, the available evidence demonstrates a rather slow growth of these small masses, with an annual size increase not greater of 0.5 cm [13–17]. Furthermore, between 15% and 30% of small renal tumours are confirmed to be benign or to have a low grade and low-malignant potential on pathological examination [18–21].

As a result, urologists now face a subset of early-stage asymptomatic patients with clinical, pathological, and morbid characteristics clearly different from those with a classically presented renal malignancy. The management of this group of patients, while still controversial, has evolved dramatically in recent years. Conservative approach by means of active monitoring or watchful waiting has been advocated by some authors [14, 22–24], and is a feasible option particularly in the elderly and significantly comorbid patient. Surgery, however, is the preferred management option for the younger, healthier patient. In recent years, nephron-sparing surgery (open and laparoscopic partial nephrectomy) has become the standard treatment for small renal masses, with data available from large series confirming similar 5-year cancer-specific survival rates (90%–100%) and a low risk (0%–3%) of local recurrence [25–29]. Although laparoscopic partial nephrectomy has clear advantages over the open approach, particularly on wound-related morbidity, its technical difficulty has limited its widespread use. Consequently, laparoscopic and percutaneous ablative techniques in renal surgery, such as, radio frequency ablation (RFA), high-intensity focused ultrasound (HIFU), and cryoablation (CA) are being increasingly utilized as they offer parenchymal preservation along with less morbidity. Although long-term oncological data is currently not available, present 5-year followup data is very encouraging. This article will focus on cryoablation (CA) of small renal masses and in particular, on laparoscopic cryoablation (LCA), with an up-to-date review of the available literature and detailed analysis of the largest published series.

## 2. HISTORICAL BACKGROUND

Cryoablation has been used in medicine since James Arnott, back in 1845–1851, demonstrated that freezing temperatures could be applied to cause tissue destruction [30]. Further interests in this field with improved delivery system and understanding of freeze-thaw sequence were followed by the use of CA in the treatment of prostate cancer only to be abandoned because of local complications [31–34].

At the turn of the last century, driven by the need for minimally invasive techniques and facilitated by rapid technological developments, a renewed interest on cryoablation and its applications in urological oncology re-emerged. Experience with vacuum-insulated liquid nitrogen or argon-cooled probes in other disciplines and technological advantages in intraoperative imaging [35], laparoscopic USS probes in particular, has allowed a safe and efficient targeting of kidney tumours. As a result, renal cryoablation, either percutaneous or laparoscopic, has become a feasible and exciting new minimally invasive surgical option for the treatment of small masses.

## 3. CRYOBIOLOGY AND PATHOPHYSIOLOGY OF CRYOABLATION

Cryoablation causes tissue destruction by a direct, as well as by a vascular, delayed mechanism [36, 37]. Direct cell damage begins with falling temperatures as structural/functional cell components are stressed and cell metabolism progressively fails. With freezing, ice crystal formation first occurs in the extracellular space, creating a hyperosmotic environment which draws water from the cells and, by a “solution-effect injury,” causes cell shrinkage and membrane damage. With further cooling, especially at high cooling rates, ice crystals will form within the cell. This phenomenon, possibly facilitated by cell-to-cell propagation via intercellular channels [38], is almost always lethal to the cell. While some cells will contain ice crystals at temperatures as high as −15°C, certainty of intracellular ice formation requires temperatures below −40°C (homogeneous nucleation) [37, 39]. During thawing, with temperatures above −40°C, ice crystals fuse into larger crystals (“recrystallization”) which, together with a transient hypotonic extracellular environment that draws water back into the cell, will result in further damage of the cell membrane and membrane rupture.

Indirectly, hypoxic damage occurs as a result of microvascular stasis. With lowering temperatures, initial vasoconstriction produces a decrease in blood flow, with complete cessation during freezing. During thawing, the circulation returns with transient vasodilatation. Endothelial damage produces increased permeability, oedema, platelet aggregation, and formation of thrombi, resulting in a sustained microvascular occlusion and stagnation [40, 41].

While downregulation of tumour suppressor genes essential to the control of apoptosis has been implicated in most malignancies and proapoptotic factors such as hypothermia, ischaemia, inflammation, elevated calcium levels, immunologic-based mechanisms including macrophage recruitment are associated with freezing injury. Recent studies implicate gene regulated cell death (apoptosis) in cryosurgical outcomes [42, 43].

The histological end result is a confluent coagulative necrosis, as evidenced by the presence of numerous histiocytes, cholesterol crystals, and dystrophic calcification within the cryolesion, with eventual fibrosis and scarring. Features that have been demonstrated in animal models [44, 45] as well as in human renal cryoablated tumours [46, 47].

## 4. TECHNICAL PRINCIPLES OF CRYOABLATION

Renal cryoablation has been shown to produce predictable and reproducible tissue destruction in animal models [48–53]. Cell damage depends on the cooling rate, the number of freeze-thaw cycles [45], the lowest temperatures achieved as well as the hold time at subzero temperatures [37, 54]. Importantly, while temperature below −19.4°C has been shown to be sufficient for complete destruction of normal renal parenchyma [48], neoplastic cells may require temperatures as low as −50°C to guarantee cell death [37]. Moreover, preclinical models have demonstrated that such low temperatures can only be achieved within a core volume of tissue, limited to 4 to 6 mm inside the edge of the forming ice ball [48, 49]. Thus, most authors will extend the ice ball to 1 cm beyond the tumour margins, incorporating the outer few millimetres or “indeterminate zone” and a margin of normal renal parenchyma, to optimize oncological control [55].

Modern cryoprobes can achieve temperatures as low as −190°C by exploiting the Joule-Thompson effect. Typically, compressed argon gas is allowed to expand through a small orifice, producing temperatures well below those required to ablate normal renal tissue (−19.4°C) [48] and cancer cells (−40°C), as demonstrated on in vivo prostatic [56] and renal cryolesions [45]. Although, the number of cycles is still controversial, early data from in vivo experiments [37] has now been corroborated in cryoablated tumours. With the incorporation of double-freeze cycles, a larger cryolesion can be achieved than with a single cycle. Apart of the number of cycles and in contrast to original experimental observations, it has been demonstrated that rapid thawing, with helium gas at 15°C to 20°C/min, does not infringe on lesion size, while reducing procedural time [45].

## 5. CLINICAL APPLICATION OF RENAL CRYOABLATION

Following the first experimental renal cryosurgery by Lutzeyer et al. [57, 58], it was not until 1995 that Uchida et al. performed the first reported percutaneous cryoablation in canine kidneys and, later that year, reproduced the technique in 2 patients with advanced renal carcinoma [59]. CA has developed rapidly since and can currently be delivered via open, laparoscopic and percutaneous approaches.

## 6. OPEN CA

Feasibility of open renal cryotherapy in humans was first reported in 1996 by Delworth et al., at the University of Texas M. D. Anderson Cancer Center, after a successful treatment of two patients with tumours in a solitary kidney, one renal cell carcinoma and one angiomyolipoma [60]. Rukstalis et al. published in 2001 the first report on systematic use of this approach [61]. A total of 29 tumours (22 solid masses and 7 complex cysts) with a median size of 2.2 cm were treated using intraoperative ultrasound monitoring and double-freeze sequences. With a median followup of 16 months, only one patient had a biopsy-confirmed recurrent tumour. Five serious adverse events occurred in 5 patients, with only one event directly related to the procedure. Overall, 91.3% of patients demonstrated a complete radiographic response [61]. In 2002, Khorsandi et al. reported open cryoablation on 17 patients with small renal tumours (median 2 cm; range: 1.1–4.2 cm), using a double freeze-thaw technique to −180°C. Median age was 62 years (range: 35–75 years). With a median followup of 30 months (range: 10–60 months), MRI demonstrated infarction and a reduction of lesion size in 15 of 16 cases. One patient's mass was unchanged at 3 months followup [62].

Whilst open CA offers safe parenchymal preservation, wound morbidity appears to be the drawback of this technique. With only two further reports in the literature [63, 64], practice in recent years has clearly favoured the laparoscopic and percutaneous approaches, with a marked trend towards the former.

## 7. LAPAROSCOPIC CA (LCA)

Laparoscopic cryoablation (LCA) offers several procedural advantages, namely, a minimally invasive approach, magnification, direct visualization of the tumour and internal manipulation of the cryoprobes and dual (visual and ultrasound) monitoring of the cryolesion [65] as well as allowing extensive pathologic sampling [66]. Surgeon preference and experience are crucial for choosing between transperitoneal and retroperineoscopic approaches. While transperitoneal approach allows a more direct access to anterior tumours, it carries a higher risk of bowel injury. Posteriorly located tumours are more amenable to retroperineoscopy, however, blunt dissection in this approach is associated with an increased risk of bleeding [12].

**Figure 1:**
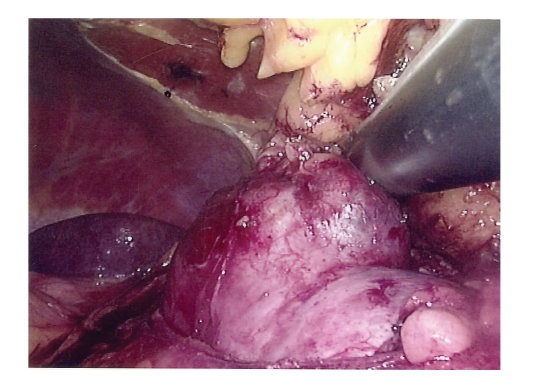
Ultrasound scanning of an exophytic left renal tumour exposed by laparoscopic mobilisation prior to cryoablation.

**Figure 2:**
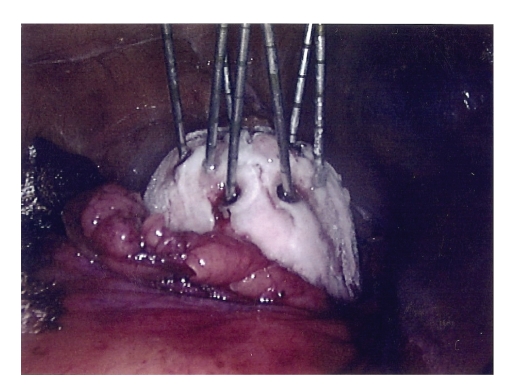
Visualisation of the Ice-ball during thawing, demonstrating arrangement of cryoprobes and temperature monitoring probes.

In our experience at Sunderland Royal Hospital, from September 2005, 17 patients have undergone LCA under a strict departmental protocol. Patient is positioned in lateral position as for nephrectomy. We used one 10 mm port for camera and two working ports (10 and 5 mm). Depending on the position of the tumour, we have used a further 5 mm port to retract the liver. Following adequate pneumoperitoneum, kidney is mobilised in order to access the tumour favourably for the needle insertion and for ultrasound probe positioning. Gerota's fascia and peri-renal fat are carefully dissected to expose the tumour. A standard biopsy of the tumour is then performed. Cryoprobes (17G) are inserted under visual and ultrasound control (Figure 1), at a maximum distance of 1 cm apart from each other. Tumour core temperature and tumour margin temperature are monitored throughout. Our protocol includes 2 Freeze-Thaw cycles: Freezing, during 10 minutes, achieving a core temperature of −70°C and a peripheral temperature of at least −40°C, followed by of 10 minutes of thawing (5 minutes active + 5 minutes passive thawing). The ice-ball is monitored visually by the surgeon and by real-time laparoscopic USS probe (Hitachi) performed by an expert consultant uroradiologist (Figure 2). The ice-ball is extended to a minimum of 5 mm beyond the tumour margins. Following surgery, our preferred imaging modality is pre- and postcontrast CT, which is performed as part of our followup protocol at 3, 6, 12, 18, 24 and yearly thereafter. Renal function is checked at each clinic visit. Since majority of recurrences are found at 3 months and almost all at 1 year, CT or MRI at 3, 6, 12 months and yearly thereafter has been recommended by other authors [67].

No treatment failures have been so far observed. Twelve masses (70%) were demonstrated to be a RCC. Histology in one patient revealed urothelial carcinoma necessitating nephroureterectomy. One patient required transfusion and another underwent embolisation of an arterio-venous fistula.

A comprehensive review of the literature reveals promising results. A summary of outcomes for the larger series is summarised in Table 1.

In 2003, Lee et al. reported results of LCA with ultrasound guidance, double-freeze cycle and up to 3-years followup (mean 20.25 months), in 20 patients with small renal masses (1.4–4.5 cm) and age ranging from 43 to 84 years. Mean operating time was 305.9 minutes and blood loss 92.5 mL (50–200 mL). Biopsies demonstrated renal cell carcinoma (RCC) in 11 cases, none of which had recurred. Overall survival was 100% for this cohort [68].

In the same year, Nadler et al. reported results on 15 patients. Mean age was 68.5 years (range: 49–86 years). Mean tumour size was 2.15 cm (range: 1.2–3.2 cm), and mean estimated blood loss was 67 mL (range: 15–125 mL). RCC was demonstrated in 10 cases. Median radiographic followup (15 months, range 4.9–27 months) revealed stable lesions in all patients. There was 1 treatment failure due to incomplete treatment of the periphery of the lesion. Another patient, with a successfully treated tumour, had a positive followup biopsy due to multifocal papillary renal cell carcinoma and required nephrectomy [69].

Initial data from the Southern Illinois University was published in 2005, a total of 25 patients with an average age of 65 years (range: 32–83 years) and mean tumour size of 2.4 cm (range: 1.5–3.6 cm). Pathology revealed RCC in 72% of cases. With a followup for up to 36 months (range: 6–36 months), no recurrences were reported [70]. Subsequent publication including 84 consecutive patients with an average age of 67 years and a mean tumour size of 2.6 cm (range: 1.2-4.7 cm) of which, 70 procedures were performed laparoscopically. They reported 7 conversions, 2 of them for failures. Intraoperative biopsy yielded a 59% malignancy rate. With a mean followup of 10 months (range: 3–36 months), an abnormal postoperative enhancement occurred in 2 patients, one of which was confirmed to be a RCC [71].

Cestari et al. presented data from a cohort of 70 patients treated with laparoscopic (48 transperitoneal, 28 retroperineoscopic) cryoablation (LCA). Average age was 63.2 years, mean size 2.37 cm (range: 1–6 cm), mean operating time and blood loss were 181.4 minutes and 164.2 mL, respectively. With a followup of up to 36 months, progressive reduction of the cryolesion was demonstrated in all patients on MRI. Only 1 patient required radical nephrectomy for recurrent tumour [72].

In 2005, with 168 cases performed at the Cleveland Clinic Foundation (1997–2005), Hegarty et al. reported, prospectively collected, intermediate-term (3 years) followup data in 56 patients, with a mean tumour size of 2.3 cm, who underwent LCA under a strict MRI imaging and CT-guided biopsy followup protocol, introduced in 1997. Sequential mean cryolesion size on MRI on postoperative 1 day, at 3 and 6 months, and at 1, 2, and 3 years was 3.7, 2.8, 2.3, 1.7, 1.2, and 0.9 cm, representing a 26%, 39%, 56%, 69%, and 75% reduction in cryolesion size at 3 and 6 months and 1, 2, and 3 years, respectively. At 3 years, 17 cryolesions (38%) had completely disappeared on MRI. Postoperative needle biopsy identified locally persistent/recurrent renal tumour in 2 patients. In the 51 patients undergoing cryotherapy for a unilateral, sporadic renal tumour 3-year cancer specific survival was 98%. There was no open conversion. During the 2006 AUA Meeting, this group presented updated results on 60 patients that had each completed 5 years followup (median 72 months). Mean tumour size was 2.3 cm (range 1–4.5 cm). Three patients (6.7%) developed local recurrence. Overall and cancer-specific 5-year survival was 82% and 100%, respectively [73].

Moon et al. published results on 16 patients with small renal masses (mean size 2.6 cm), and their mean operating time was 188 minutes. There was 1 reported conversion, and mean blood loss was 40 mL. Tumour biopsy demonstrated 5 RCC. With a mean followup of 9.6 months, all tumours remained nonenhancing and either stable or smaller than the original lesion [74]. This group has recently reported combined data from its 5-year experience with renal cryoablation on 88 cases, treated by LCA [58] or PCA [20]. Mean tumour size was 2.6 cm. At a mean followup of 19 months, the overall, cancer-specific and recurrence-free survival rates were 88.5%, 100%, and 98.7%, respectively. Four patients required a further treatment due to persistent disease, and one had progression to locally advanced disease [75].

In 2007 Polascik et al. published results from his experience in 26 patients who underwent LCA using third-generation cryotechnology, for 28 renal masses of 3.5 cm or less (median 2 cm). Patients were followed by serial CT or MRI scan, at least every six months after cryoablation. The mean patient age was 64 years (range: 44–79), and the mean followup was 20.9 months. The median tumour size was 2.0 cm (range: 1–3.5 cm). No patient was converted to open surgery. With an overall survival rate of 100%, no evidence of recurrence or progression was found in this cohort [76].

With 47 cases in their series, Beemster et al., from the University of Amsterdam group, have now published data on 26 patients with available followup of 6 months or more. With an average followup of 17.2 months (range: 6–36 months) and a mean tumour size of 2.4 cm (range: 1.3–3.8 cm), only 1 treatment failure has been reported [77].

In agreement with data generated by larger series, preliminary results from smaller series have recently been published [78–81]. Although comprising smaller number of patients and limited followup in some cases, the published series clearly demonstrate the increasing interest and rapid expansion of this novel ablative technique.

## 8. PERCUTANEOUS CRYOABLATION (PCA)

While technical limitations hampered initial attempts at percutaneous cryoablation in human kidneys [59], the rapid development of argon technology and ultrathin probes, together with CT and open access interventional MRI, allowing real-time monitoring of the ice ball, provided the much needed technical breakthroughs, making this approach safe and reproducible.

In 2001, Shingleton and Sewell [82] reported their initial experience in 20 patients (22 tumours) treated with 2 or 3 mm cryoprobes and interventional MRI. Mean tumour size was 3 cm (range: 1.8–7 cm), and average treatment time was 97 minutes (range: 56–172 minutes). Procedures were performed under general anaesthesia or sedation, and 95% of patients were discharged within 24 hours. With a mean followup of 9.1 months (range: 3–14 months), they reported only one failure, requiring retreatment. The only complication was a superficial wound abscess. Recently, the authors have updated their series including patients with von Hipple-Lindau [83] and with tumour/s in a solitary kidney [84]. With an average followup of 24 months, 9 (15%) cases required retreatment due to incomplete initial ablation. Only 1 patient required transfusion, and there were no reported cancer-related deaths.

Experience on 23 patients (26 tumours) with mean size 2.6 cm (range: 1–4.6 cm) and mean age of 66 years (range: 43–86 years) was reported by Silverman et al., using a 0.5-T open MR imaging system and general anesthesia. Twenty four masses were RCCs, 1 was an urothelial carcinoma and 1 was an angiomyolipoma. With a mean followup of 14 months (range: 4–30 months), 24/26 tumours were successfully ablated, 23 of which required only one treatment session. In 2 cases, a small enhancing nodule located at the margin proved to be recurrent tumours. Two complications (1 haemorrhage requiring transfusion and 1 abscess drained percutaneously) occurred in a total of 27 cryoablations [85].

In 2006 Gupta et al. published CT-guided PCA on 27 tumours of 5 cm or less (mean size 2.5 cm), using conscious sedation and real-time CT monitoring. With 1 month or more followup imaging available on 16 cases (mean 5.9 months), 15 tumours showed no signs of enhancement. In 1 case, blood transfusion was required for bleeding [86].

The Mayo Clinic experience on 40 cases of PCA with CT monitoring has recently been published [87]. Mean tumour size was 4.2 cm (range: 3.0–7.2 cm) and at least 3 months followup was available in 65% of the cases (mean 9 months; range: 3–22 months). Technical success, defined as extension of the ice ball beyond the tumour margin and absence of postablation enhancement on CT, was reported in 38 (95%) cases, with no tumour recurrence or progression in the cohort. Overall complication rate for this cohort was reported at 8%.

## 9. FUTURE DIRECTIONS

Initial studies of combination therapy with 5-FU prior to freezing, indicated a temperature-dependent reduction on cell viability in a prostate cancer cell (PC-3) model [88]. Furthermore, molecular analysis using this model has demonstrated a synergistic effect of sublethal concentrations of 5-FU and Cisplatin prior to freezing (−15°C), mediated by a shift in the Bcl-2 to Bax ratio to a prodeath tendency [89]. Similar synergistic response has been reported in a renal cell model, the data suggesting that 5-FU chemotherapy may be more effective when followed by cryosurgery [90]. In the clinical setting, synergistic activity of cryoablation and cyclophosphamide is currently been evaluated on advanced epithelial tumours (NCI. Trial protocol NCT00499733).

Equally, since freezing enhances the radiosentitivity of cells, combination of radiotherapy with cryoablation may potentially confer benefits [65], as already indicated in preclinical models of prostate cancer, where adjuvant radiation and curcumin have demonstrated a synergistic effect with cryoablation [91].

At the time of writing this review, the Cleveland Clinic group have made public the initial results employing Single Port Access Renal Cryoablation (SPARC). A total of 6 patients, with mean tumour size of 2.6 ± 0.4 cm, successfully underwent SPARC, via a transperitoneal or retroperitoneal approach, with no intraoperative complications and no need for conversion, demonstrating the feasibility and safety of this, potentially scarless, procedure [92, 93].

Further development of imaging techniques and cryoprobe technology, clinical evaluation of combination therapy with conventional chemo- and radiotherapy, together with promising novel cryoenhancers, may have major implications on the management of small renal masses in the future

## 10. CONCLUSIONS

Widespread implementation of USS, CT, and MRI has resulted in an increased detection of early, small renal masses. In the last 20 years, the proportion of incidentally found renal tumours raised from 13% to an estimated 60%, with a substantial parallel decrease in tumour stage, grade, and proportion of metastasis at presentation, in these patients [94]. As a result, urologists are now faced with a new cohort of asymptomatic, healthier patients, with incidentally found small renal masses.

While open partial nephrectomy is still the reference standard [95], its associated morbidity has encouraged researchers and practicing clinicians towards less radical approaches, thus the rapid development of laparoscopic partial nephrectomy and novel ablative techniques such as radiofrequency ablation (RFA), high-intensity focused ultrasound (HIFU), and cryoablation (CA). Among ablative techniques, cryotherapy, and in particular laparoscopic cryoablation, is the most extensively studied and the one with more rapid expansion in clinical practice.

Cryosurgery offers the clear advantage of combining a nephron-sparing surgery together with a minimally invasive approach. Anaesthetic requirements, postoperative analgesia, and hospital stay are significantly reduced, with a much rapid return to normal activity and work.

In the early days of development and clinical implementation of cryoablation, concerns were raised regarding safety of the procedure, the lack of followup, and oncological outcome [96].

Regarding the safety of the procedure, published studies up to this day have shown minimal procedure-specific morbidity, with complication rates comparable or better than current available minimally invasive procedures. Reports from the largest series have demonstrated to be a less morbid procedure than laparoscopic partial nephrectomy, with a comparable 5-year oncological safety [97].

Among the novel ablative techniques, radio frequency ablation (RFA) is the procedures with more emerging clinical data. Although the procedure-specific morbidity, mostly based on small and nonstandardised series, appears to be low, serious issues have been raised regarding the RFA cell-killing potential and its higher risk of local disease recurrence, as demonstrated en several clinical studies [98–102].

When compared to RFA, available data from preclinical [52] and several clinical studies confer to cryoablation an advantageous oncological safety profile. The Cleveland Clinic group have recently published results from their RFA and LCA series, highlighting the issue of residual disease and demonstrating a clear advantage in the LCA cohort. With 109 renal lesions (88 patients) treated with RFA and 192 lesions (176 patients) treated with LCA, radiographic (CT or MRI) success at 6 months was 85% and 90% for RFA and LCA, respectively. More importantly, when lesions were later biopsied at 6 months, the success in the RFA cohort decreased to 64.8%, while LCA success remained high at 93.8%. Six of 13 patients (46.2%) with a 6-month positive biopsy after radio frequency ablation demonstrated no enhancement on posttreatment MRI or CT, while in the LCA group, all positive biopsies revealed posttreatment enhancement on imaging just before biopsy. The authors recommend postradio frequency ablation followup biopsy due to the significant risk of residual renal cell cancer without radiographic evidence [103].

Supporting these findings, a recent meta-analysis of available data demonstrates a higher risk of local disease recurrence in tumours treated with RFA, when compared to those managed by cryoablation [104].

While long-term followup is still awaited, encouraging results have been reported in series with up to 5-year followup, with cancer-specific survival rates ranging from 98 to 100% [68, 70, 72, 73, 76, 105] with LCA and 97% with PCA [7]. This is comparable to 5-year cancer-specific survival rate of 92%, reported with partial nephrectomy [95, 97, 106].

While clinical application and indications of cryoablation of small renal masses are still not clearly defined, it is recommended by available clinical evidence, that CA should be reserved for small (<3 cm) solid-enhancing renal masses in older patients with high operative risk. Young age, tumour size >4 cm, hilar tumours, intrarenal tumours, and cystic lesions can be regarded as relative contraindication, whilst irreversible coagulopathy is widely accepted as an absolute contraindication [107].

## Figures and Tables

**Table 1:** Summary of largest reported series on LCA.

	*n*	Age, years	Follow-up, months	Tumour diameter, cm	% of RCC	Failures/ Recurrences	Operative time, min.	Hospital stay, days	Complications
Lee et al. [68]	**20**	67.9 (43–84)	20.3 (1–40)	2.6 (1.4–4.5)	55%	1/0	305.9	2.6	Atrial fibrilation (1), ECG changes, no MI (1), Pancreatic injury (1), transient raised lipase-amylase (5), Transfusion (1)

Nadler et al. [69]	**15**	68.5 (49–86)	15 (4.9–27)	2.15 (1.2–3.2)	67%	1/1		3.5	Respiratory failure (1), prolonged ileus (1)

Schwartz et al.** ^§^ [71]	**70**	67 (32–85)	10 (3–36)	2.6 (1.2–4.7)	59%	1/1		2.2	CVA (1), transfusion (2), renal fracture (1), Transient hydronephrosis (1)

Cestari et al. [72]	**70**	63.2	36 (28–48)	2.37 (1–6)	69%	0/1	181.4	4.5	Haematuria (2), pyrexia (6), bleeding (1), Anaemia (6), Pulmonar oedema (1), PUJ Obstruction

Hegarty et al. [73]	**60**		72	2.3 (1–4.5)		0/3	174.2	2.4	2% transfusion rate. Congestive Heart Failure (1), Splenic haematoma (1), oesophagitis (1), Pleural effusion (1)

Moon et al. [74]	**16**		9.6 (1–28)	2.6 (1.5–3.5)	33%	0/0	188	1.9	Pneumonia (1)

Polascik et al. [76]	**26**	64 (44–79)	20.9 (2–53)	2.5 (1–3.5)		0/0		2 (0–9)*	Transfusion (1), prolonged ileus (1)

Beemster et al. [77]	**26**		17.2 (6–36)	2.4 (1.3–3.8)		1/0			Paraesthesia (1), UTI (1), pneumonia

*n*: Number of patients. RCC: Renal cell carcinomas found on histology. Values expressed as mean unless stated otherwise. ^§^Total of 84 cases in this series. Only 70 of them were performed laparoscopically. *Value expressed as median.
